# The Physical Health Care Fidelity Scale: Psychometric Properties

**DOI:** 10.1007/s10488-020-01019-0

**Published:** 2020-02-08

**Authors:** Torleif Ruud, Tordis Sørensen Høifødt, Delia Cimpean Hendrick, Robert E. Drake, Anne Høye, Matthew Landers, Kristin S. Heiervang, Gary R. Bond

**Affiliations:** 1 Division of Mental Health Services, Akershus University Hospital, Lørenskog, Norway; 2 Institute of Clinical Medicine, University of Oslo, Oslo, Norway; 3 University Hospital Northern Norway, Tromsø, Norway; 4 Institute of Clincial Medicine, UiT The Arctic University of Norway, Tromsø, Norway; 5 WestBridge, Manchester, NH USA; 6 Westat, Lebanon, NH USA; 7 Duke University, Durham, NC USA

**Keywords:** Psychoses, Physical health care, Evidence-based practice, Implementation, Fidelity scale

## Abstract

Mental health programs need an instrument to monitor adherence to evidence-based physical health care for people with serious mental illness. The paper describes the Physical Health Care Fidelity Scale and study interrater reliability, frequency distribution, sensitivity to change and feasibility. Four fidelity assessments were conducted over 18 months at 13 sites randomized to implementation support for evidence-based physical health care. We found good to excellent interrater reliability, adequate sensitivity for change, good feasibility and wide variability in fidelity across sites after 18 months of implementation. Programs were more successful in establishing Policies stating physical health care standards than in implementing these Policies. The Physical Health Care Fidelity Scale measures and guides implementation of evidence-based physical health care reliably.

**Trial registration**: ClinicalTrials.gov Identifier: NCT03271242

**Electronic supplementary material:**

The online version of this article (10.1007/s10488-020-01019-0) contains supplementary material, which is available to authorized users.

## Introduction

Physical disorders account for high morbidity, high mortality and earlier death for persons with psychosis, compared to the general population (Chang et al. 2011; Heiberg et al. 2018; Hjorthoj et al. 2017; Kilbourne et al. 2009; Lawrence et al. 2013; Nordentoft et al. 2013; Osby et al. 2016; Saha et al. 2007). A major part of this is due to cardiovascular and metabolic disorders including heart disease, hypertension, diabetes, hyperlipidemia and obesity (Correll et al. 2014; Foguet-Boreu et al. 2016; Stubbs et al. 2015; Vancampfort et al. 2016). Smoking, unhealthy diet, low physical activity and low medical and dental care contribute to physical illnesses and shorter lives (Correll et al. 2017; Kisely et al. 2015a, b; Vancampfort et al. 2015; Wey et al. 2016). Antipsychotic medications may add to the physical health burden by producing side effects like obesity, metabolic disorders, and cardiac disease as well as by negatively affecting dental health (Kahl, 2018; Kisely et al. 2015a, b; Tek et al. 2016).

Based on the needs for improvement of physical health and physical health care for people with psychosis, clinicians and researchers have developed evidence-based interventions for many aspects of physical health for these patients. However, to integrate and implement these interventions in routine clinical practice, there is a need for a cohesive model where these interventions are combined. Several of the evidence-based interventions or components have been combined in models for cardiometabolic risk management (Curtis et al. 2012), which have been introduced in some countries, including Norway. In a section below in methods we describe briefly such evidence-based components of physical health care for people with psychosis and give references to evidence for these components.

Clinical guidelines are available, but implementation is typically fragmented or lacking in daily clinical work (Barbui et al. 2014; Citrome and Yeomans 2005; Tansella and Thornicroft 2009; Weinmann et al. 2007). In spite of these findings, we found no fidelity scale measuring these evidence-based interventions. The dearth of extant fidelity scales in this area suggests a great need for a psychometrically valid fidelity scale for physical health care.

### Aims

The aims of this study were to define a comprehensive model of physical health care for people with psychosis consisting of evidence-based components, and to develop a fidelity scale measuring physical health care and study its psychometric properties, including interrater reliability, frequency distribution, sensitivity to change and feasibility.

## Methods

### Overview

Development of the Physical Health Care Fidelity Scale and testing its psychometric properties were part of a study on implementation of four evidence-based practices for treatment of patients with psychoses in mental health services (ClinicalTrials NCT03271242). Thirteen sites from five health trusts in Norway were randomized to receive implementation support to implement evidence-based physical health care. The current paper reports the findings of a secondary data analysis of physical health care fidelity assessments at these 13 sites. Prior to the study, all sites were providing physical health care, but without support for following evidence-based guidelines. The Regional Committee for Medical and Health Research Ethics approved the study (REK 2015/2169), which followed the principles in the Declaration of Helsinki.

### Defining Evidence-Based Physical Health Care for People with Psychosis

Based on the research literature we identified five evidence-based components of physical health care for people with psychosis. These are briefly listed below and in Table 1 with some key references.

**Table 1:** Evidence for components of evidence-based practice for physical health care

Component of evidence-based physical health care	Scale items	Key references to evidence
Policy and procedures promoting and supporting physical fitness	1, 2, 7	Chacón et al. (2011), Dauwan et al. (2016) and Firth et al. (2015)
Policy and procedures monitoring cardiovascular risk factors and treating physical illnesses	6, 8, 9, 11, 12, 13, 14	Ayerbe et al. (2018), Correll et al. (2014), De Hert et al. (2011, 2009), Foguet-Boreu et al. (2016), Laursen et al. (2014) and Mitchell et al. (2015)
Policy and procedures promoting and supporting healthy diet	3, 10	Singh et al. (2018) and Teasdale et al. (2016)
Policy and procedures promoting and supporting smoking cessation	4, 16	Banham and Gilbody (2010) and Jahagirdar and Kaunelis (2017)
Policy and procedures promoting and supporting dental and oral health	5, 17	Khokhar et al. (2016) and Kisely et al. (2015a, b)

#### Promotion and Support of Physical Fitness

Lifestyle interventions based on diet and exercise have been documented to reduce the negative impact of cardiovascular risk (Chacón et al. 2011) and improve clinical symptoms, quality of life, global functioning and depressive symptoms in patients with schizophrenia (Dauwan et al. 2016). Implementation of a sufficient dose of exercise can be feasible and effective interventions and improve functioning, co-morbid disorders and neurocognition (Firth et al. 2015).

#### Monitoring Cardiovascular Risk Factors and Treatment of Physical Illness

People with psychosis have increased risk of cardiovascular illness (Foguet-Boreu et al. 2016) including in early phases of the illness (Correll et al. 2014). But they are less likely than others to receive appropriate health care (Ayerbe et al. 2018; De Hert et al. 2011; Laursen et al. 2014; Mitchell et al. 2015). Mental health care and primary care must collaborate to improve monitoring and physical health care for people with psychosis (De Hert et al. 2009).

#### Promotion and Support of Healthy Diet

Dietary education and counselling have been shown to contribute to weight loss or preventing obesity in persons with psychosis (Singh et al. 2018). Individual dietetic consultations combined with group classes with shopping and cooking have been found to promote healthy diets in young people with first time psychosis (Teasdale et al. 2016).

#### Promotion and Support of Smoking Cessation

Treatment of tobacco dependence is equally feasible and effective in people with psychosis as in the general population, and it does not worsen mental state (Banham and Gilbody 2010). Effective treatments include nicotine replacement therapy (Jahagirdar and Kaunelis 2017).

#### Promotion and Support of Dental and Oral Health

Physical health care for people with psychosis should encompass oral health assessment, help with oral hygiene and early dental referral (Kisely et al. 2015a, b). Evidence for the effectiveness of oral health education, and practical support to visits dentists and brush teeth is limited (Khokhar et al. 2016).

### Development of the Physical Health Care Fidelity Scale

Following standardized procedures for fidelity scale development (Bond et al. 2000), we identified five core components of evidence-based physical health care for persons with psychosis from current research reviews. Table 1 shows these components, the related items in the fidelity scale, and key references documenting evidence. For each component we defined two or more items, and for each item we defined operationalized criteria and rules for rating each item on five steps from no to full fidelity. We asked some clinicians and researchers for comments on this draft version of the fidelity scale, and then made final adjustments based on their input and on informal pilot testing in some sites. While a comprehensive treatment for physical health care also addresses substance use, we did not include substance treatment in the current scale because a separate fidelity scale already measures integrated dual disorders treatment (Chandler 2011).

### Sites

The sample consisted of 13 sites from five health trusts in urban and rural areas throughout Norway. Six of the sites were teams in community mental health centers and seven were inpatient wards for patients with psychosis. All these teams and wards in the specialized mental health services had assessment and treatment of people with psychosis as a major task, but also general hospital clinics and primary health and social care are serving this patient group. The general practitioners (GPs) often have a role in coordinating the total health care for the patient.

### Procedures

The sites received training and support to help implementation. Approximately 130 mental health professionals (an average of 10 leaders and clinicians from each site) participated in a one-day workshop led by Norwegian experts on physical health care for persons with psychosis. The research team also developed the Toolkit of Physical Health Care and distributed it to the sites at the launch of the project (Høifødt and Høye 2016). The toolkit included a description of each component of an evidence-based physical health care with rationale and references, description of clinical details including the algorithm for cardiometabolic risk management developed by Curtis et al. (2012), key literature, presentations from the workshop, the fidelity scale, and patient information for clinical use. Implementation trainers offered in-person implementation support biweekly for 6 months and then monthly for an additional 12 months, and the sites used this actively most of the time.

A pair of two trained fidelity assessors, independent from the clinical staff and using fidelity guidelines, conducted assessments and provided feedback to each site at baseline, and after 6, 12, and 18 months. A group of 15 researchers (psychologists, psychiatrists, nurses and other health professionals) served as assessors, and the two fidelity assessors varied partly across sites and assessment periods. The assessors conducted interviews with leaders and clinicians, reviewed written documentation of Policies and Procedures, and reviewed 10 randomly selected patient records. They made independent fidelity ratings, compared ratings, resolved discrepancies through discussion to reach consensus, and recorded independent and consensus ratings.

### Measures

#### The Physical Health Care Fidelity Scale

The fidelity scale includes 17 items measuring five components of evidence-based physical health care, as shown in Table 1. Each item is rated on a 5-point behaviorally anchored rating scale, have 3–7 specific criteria and rules for rating based on number of criteria met. The total scale includes two subscales: Policies (6 items, Items 1–6) and Practices (11 items, Items 7–17). Fidelity assessors rated the Policies items based on semi-structured interviews with leaders and key clinicians, and on reviewing written Policies or Procedures. The assessors rated the Practices items based on information in 10 randomly selected patient records, including progress notes and prescription orders over the previous 3 months for inpatients and the previous 6 months for outpatients. For these items they used a summary sheet and made dichotomous ratings for each patient record on the 3–7 specific criteria for each item, and then made a fidelity rating for each item based in number of patient records passed. The scoring of the subscales and total fidelity scale represented the unweighted sum of the item ratings divided by the number of items. The fidelity scale with instructions is available as an online appendix. Table 2 contains abbreviated names of items.

**Table 2:** Percentage exact agreement and interrater reliability* for items based on two raters’ rating independently 13 sites 4 times for items 1–6 and altogether 95 patient records for items 7–17

Item	Short item titles	Agreement (%)	ICC*	Kappa*
	*Policies Subscale items*			
1.	Policy promoting physical fitness	65	.90	
2.	Practical help to physical activities	81	.88	
3.	Policy supporting healthy diet	69	.88	
4.	Policy supporting smoking cessation	62	.89	
5.	Policy supporting dental health	79	.92	
6.	Collaboration and communication with GP	77	.85	
	**Policies Subscale items average**	**72**	**.89**	
	*Practicies Subscale items*			
7.	Support for regular physical activities	96		.80
8.	Monitoring of physical health conditions	93		.85
9.	Documented collaboration with GP	84		.66
10.	Documented support for healthy diets	96		.58
11.	Monitoring BMI and waist circumference	99		.90
12.	Assessment and treatment of obesity/malnutrition	89		.64
13.	Assessment and treatment of hypertension	93		.85
14.	Assessment and regulation of blood sugar	89		.79
15.	Assessment and regulation of blood lipids	94		.87
16.	Interventions for smoking cessation	93		.66
17.	Monitoring of dental health	98		.85
	**Practices Subscale items average**	**93**		**.77**
	**Average for all items**	**86**		

Names and values of scales are shown in bold

*GP* general practitioner

*Intraclass correlation (ICC) for items rated 1–5 and Cohen’s kappa for patient records rated passed/failed

#### Feasibility Survey

After the final assessments, the fidelity assessors completed an online survey on their experiences with the fidelity scale. The survey included questions on whether the scale was clearly set out and had good instructions, whether necessary information was easy to find, whether the scale was easy to rate, and on how useful various sources of information were.

### Data Analyses

At each assessment, two fidelity assessors made independent fidelity ratings on the Policies items (Items 1–6), resulting in 52 assessments (13 sites each rated 4 times). To assess interrater reliability on these items and the Policies Subscale we calculated the intraclass correlation coefficient (ICC) (McGraw and Wong 1996) based on a one-way random effects analysis of variance model for agreement between two assessors. For ICC defined as above, we interpreted degree of interrater reliability as suggested by Koo and Li (2016) with the levels poor (below .50), moderate (.50 to .74), good (.75 to .90) and excellent (above .90). We also calculated percentage exact agreement for the items.

The fidelity assessors did not make independent ratings for the Practices items (Items 7–17). Instead, in order to determine interrater reliability, the assessors independently rated a subset of patient records at each fidelity site visit. From the 52 fidelity assessments we obtained independent dichotomous judgments (passed/failed for each item) for 95 patient records (usually 2 at each site visit) reviewed independently by both assessors. The two assessors divided the other 8 randomly selected patient records between them to save time and still obtain ratings on 10 patient records. Based on the 95 pairs of independent ratings of patient records we calculated percentage of exact agreement and Cohen’s kappa for the 11 Practices items. For kappa we interpreted the degree of interrater reliability as suggested in the guidelines by Cicchetti (1994) with the levels poor (below .40), fair (.40 to .59), good (.60 to .74) and excellent (.75 and above). We also calculated percentage of exact agreement and kappa on the 3–7 criteria for each of these items (See Online Appendix, Table 4).

After assessing interrater agreement and reliability, we used consensus ratings in all subsequent analyses. To estimate internal consistency of the two subscales and the total scale, we used Cronbach’s alpha, calculating an alpha coefficient for each assessment period (baseline, 6, 12, and months). For alpha we interpreted the degree of internal consistency as suggested in the guidelines by Cicchetti (1994) with the levels unacceptable (below .70), fair (.70 to .79), good (.80 to .89) and excellent (.90 and above).

We next examined the item distributions at 18 months, including mean, standard deviation, and distribution of scores across sites for full (rating = 5), adequate (4), and poor (1–3) fidelity. We also examined the distribution of site scores at 18 months. Distribution on passed/failed for criteria of all items are also reported (See Online Appendix, Table 5).

Next, we examined the longitudinal pattern of fidelity graphically and statistically for the total scale and the two subscales. We examined the pattern in change over time using one-way ANOVA repeated measures with pairwise post hoc tests with Bonferroni corrections between baseline and 6 months, and between 6 and 18 months. We also analyzed sensitivity to change in fidelity from baseline to 18 months using paired t-tests for each item, the total scale and the two subscales, including reporting means and standard deviations at baseline and 18 months. Change over time was estimated by calculating the standardized mean difference effect size (Cohen’s d_z_) for within-subjects design (Lakens 2013). We interpreted the sensitivity to change as adequate if the improvement was statistically significant and with at least a moderate effect size (Cohen’s d_z_ $\ge$ .50).

Finally, we calculated the Pearson correlation coefficient between the Policies Subscale and the Practices Subscale across the sites for each of the four times of assessment. We interpreted the correlation coefficients according to guidelines suggested by Schober et al. (2018).

From the feasibility survey we determined time the fidelity assessors on average spent on a fidelity visit, and their experiences with using the fidelity scale. We are not aware of any established measure for feasibility, but we interpreted feasibility to be good for a scale quality (clearly set out, easy to get information, easy to rate, good instruction) if more than 60% of the fidelity assessors rated agreed or agreed strongly to it in the feasibility survey. All data analyses used SPSS version 25 (https://www.ibm.com/analytics/spss-statistics-software).

## Results

### Interrater Reliability

Table 2 shows the percentage of exact agreement and interrater reliability for all items. The percentage exact agreement was on average 72% for the six Policies Subscale items, on average 93% for the 11 Practices Subscale items and on average 86% for all 17 items. The ICC was excellent for the Policies Subscale (.95) and one subscale item (.92), and good (range .85 to .90) for five items. For the 11 Practices Subscale items, kappas were excellent (.75 to .90) for seven items, good (.64 to .66) for three and fair (.58) for one. The percentage exact agreement and interrater reliability for the criteria for all items are reported in detail in Table 4 in the online appendix, which shows that the exact agreement was 80% or above for 66 (92%) of the 72 criteria. For the 25 criteria of the Policies Subscale items, kappa was excellent (.75 to 1.00) for 13 items, good (.65 to .74) for seven and fair (.46 to .57) for five. For the 47 criteria of the Practices Subscale items, kappa was excellent (.75 to .96) for 20 items, good (.61 to .74) for 15, fair (.42 to .58) for seven and poor (− .01 to .39) for five.

### Frequency Distribution

Table 3 shows descriptive statistics for each item, the Policies and Practices Subscales, and the total fidelity scale at baseline and 18 months. The table also shows distributions at 18 months regarding poor, adequate and full fidelity. The following seven Practice items did not achieve adequate fidelity after 18 months: support for physical activities, support for healthy diets, monitoring BMI and waist circumference, assessment and treatment of obesity/malnutrition, assessment and regulation of blood lipids, interventions for smoking cessation, and monitoring of dental health. This is shown in more detail in Table 5 (in the Online Appendix), which shows that at 18 months the criteria for the Policies items were rated passed for most sites, but that the criteria and patient records for the Practices items listed above were mostly rated failed.

**Table 3:** Descriptive statistics for items, subscales and fidelity scale (13 sites)

Fidelity scale items	0 months	18 months	Difference 0 and 18 months	Distribution of fidelity ratings at 18 months		
	Mean (SD)	Mean (SD)	Significance p (paired t-test)	Poor 1–3	Adequate 4	Full 5
*Policies Subscale items*						
1. Policy promoting physical fitness	2.38 (0.87)	3.23 (0.83)	.001	9	3	1
2. Practical help to physical activities	4.15 (1.21)	4.54 (0.78)	.054	2	2	9
3. Policy supporting healthy diet	2.31 (0.75)	3.23 (1.01)	.008	8	4	1
4. Policy supporting smoking cessation	2.00 (0.58)	3.62 (1.12)	.001	5	5	3
5. Policy supporting dental health	2.62 (0.51)	4.08 (0.86)	< .001	2	7	4
6. Collaboration and communication with GP	3.15 (0.69)	3.77 (0.60)	.025	4	8	1
**Policies Subscale fidelity**	**2.77 (0.48)**	**3.74 (0.55)**	** < .001**	**1**	**6**	**6**
*Practices Subscale items*						
7. Support for regular physical activities	1.15 (0.56)	1.85 (1.21)	.108	12	0	1
8. Monitoring physical health conditions	2.46 (1.76)	3.31 (1.38)	.059	7	3	3
9. Documented collaboration with GP	2.00 (1.41)	3.23 (1.64)	.036	6	3	4
10. Documented support for healthy diets	1.00 (0.00)	1.46 (0.52)	.008	13	0	0
11. Monitoring BMI and waist circumference	1.00 (0.00)	1.54 (0.88)	.047	12	1	0
12. Assessment/treatment of obesity/malnutrition	1.46 (0.97)	1.77 (0.73)	.337	13	0	0
13. Assessment/treatment of hypertension	2.54 (1.61)	3.15 (1.21)	.219	7	5	1
14. Assessment and regulation blood sugar	2.31 (1.49)	3.92 (1.26)	.003	5	2	6
15. Assessment and regulation blood lipids	2.00 (1.23)	3.00 (1.29)	.036	10	0	3
16. Interventions for smoking cessation	1.15 (0.38)	1.62 (1.19)	.139	12	0	1
17. Monitoring of dental health	1.00 (0.00)	1.46 (0.88)	.082	12	1	0
**Practices Subscale fidelity**	**1.64 (0.62)**	**2.39 (0.71)**	**.006**	**13**	**0**	**0**
**Total mean fidelity**	**2.04 (0.46)**	**2.87 (0.59)**	** < .001**	**13**	**0**	**0**

Names and values of scales are shown in bold

Internal consistency (Cronbach’s alpha) for the total fidelity scale was fair to excellent (range .78 to .91) for each of the four fidelity assessments, unacceptable to good (range .56 to .81) for the Policies Subscale and fair to good (range .72 to .89) for the Practices Subscale.

### Sensitivity to Change

**Fig. 1:**
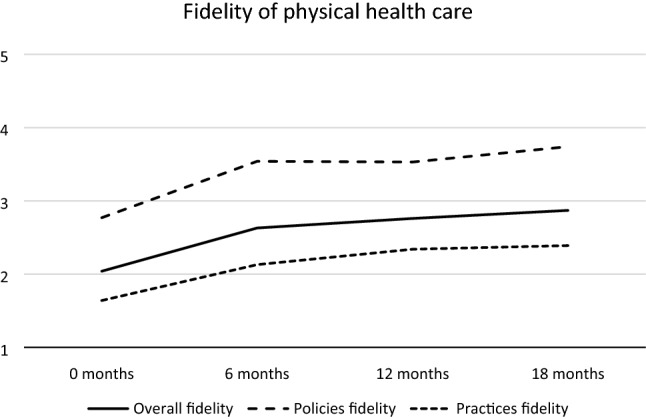
Development of overall fidelity, and at Policies and Practices fidelity

Figure 1 shows the development of fidelity across 18 months for the Policies and Practices Subscales and the total fidelity scale. The main change occurred from baseline to 6 months with little change from 6 to 18 months. At baseline, the mean site-level fidelity rating for the total scale was 2.04. By 6 months, mean fidelity had increased to 2.63, a significant increase of .59 (t = − 4.74, p = 0.001). At 18 months, fidelity was 2.87, which was a nonsignificant increase of 0.24 (t = − 1.71, p = 0.335) from 6 months. The increase of 0.83 in total fidelity from baseline to 18 months was significant (t = − 5.21, p = 0.001) and with a large effect size (Cohen’s d_z_ = 1.45). The increase was also significant for both subscales, and Cohen’s d_z_ was 1.87 for the Policies Subscale and 0.93 for the Practices Subscale. At baseline none of the sites had a total mean fidelity above 3.0, and at 18 months five sites had a fidelity above 3.0. No site achieved a mean fidelity exceeding 4.0, the benchmark for adequate fidelity, except one site at 12 months.

As shown in Fig. 1, the Policies Subscale fidelity and the Practices Subscale fidelity both showed a development parallel to the overall fidelity with the main increase during the first six months, but with the Policies Subscale fidelity above and the Practices Subscale fidelity below the overall fidelity. The Pearson correlation between the Policies Subscale fidelity and the Practices Subscale fidelity at each assessment across all sites was weak to moderate (.20, .68, .67 and .57 at baseline, 6 months, 12 months and 18 months, respectively).

### Feasibility Survey

The 15 fidelity assessors reported that they spent on average 4.6 h (SD 1.3) on a fidelity visit, including an average of 3.0 h (SD 1.0) on reading and rating patient records. Altogether 89% agreed that the scale was clearly set out, 44% that it was easy to get information, 61% that it was easy to rate, 78% that it had good instructions, and 78% that it had good instructions for preparations. Regarding data sources, assessors reported that interviews with clinicians and reading patient records were useful sources of information, while interviews with leaders were less useful.

## Discussion

The 17-item Physical Health Care Fidelity Scale operationalized evidence-based components from the research to assess the quality of physical health care. The interrater reliability (ICC) was excellent for the Policies Subscale and good to excellent for the subscale items. The interrater reliability (kappa) for the Practices Subscale items was excellent or good for all but one item. Sensitivity to change over time was adequate with significant change and large effect size for the total scale and both subscales. The feasibility was generally adequate, with the caveat that assessors reported difficulty finding some information. The distribution of site ratings at 18 months was good for half of the items, but none of the sites reached adequate fidelity level of 4.0 within 18 months. The overall picture was that the total scale and the two subscales achieved good to excellent interrater reliability, adequate sensitivity for change and good feasibility.

The interrater reliabilities (ICC) for the items and the Policy subscale were excellent. For the Practices items interrater agreement was calculated for agreement of whether specific patient records met the criteria of the item, and this was high both according to exact agreement and kappa. The interrater agreement for assessing whether criteria were met was adequate both according to exact agreement and Cohen’s kappa. Our conclusion is that the interrater reliability of the fidelity scale is adequate, and that the scale may be used for reliable assessments of fidelity to the evidence-based practice of physical health care as described in the introduction and defined by the fidelity scale. Extending the fidelity visit for two additional hours so that both assessors could review all 10 patient records and make independent fidelity rating of all items, would make it possible to calculate ICC for all items, as well as for both subscales and the total fidelity scale.

The policy items showed a reasonable distribution of ratings across sites after 18 months. Four Practice items with widely dispersed ratings at 18 months were monitoring physical illness, documented collaboration with general practitioner, monitoring hypertension and monitoring blood sugar. These medical activities are well established. By contrast, for seven Practice items for less established Practices, most sites did not achieve adequate fidelity even at 18 months. These items include supporting physical activities, healthy diets, smoking cessation and dental health, and monitoring BMI and waist circumference. The poor adherence to best Practice standards regarding monitoring obesity/malnutrition and blood lipids is concerning, but it may be that the criteria used to meet high fidelity is too stringent, indicating a need to revise calibration of these and perhaps some other items.

Undoubtedly physical health monitoring and Practice sometimes were performed well, but these interventions were not documented adequately in the patient records. But evidence-based practice includes adequate documentation. This is especially true in public mental health services where staff turnover often leads to many different medical professionals needing access to information to provide continuity of treatment. Where critical information is missing, treatment is substandard.

Inpatient mental units may provide more comprehensive physical health care and document it better than outpatient units. Moreover, some outpatients may get physical health care from a GP or in other health service, and with this care not documented in programs where they receive mental health treatment. However, for many criteria we have also included that the criterion is met if the site documents physical health care that the patient receives elsewhere, as concurrent services should keep each other informed. Such issues will be analyzed and discussed in a later paper.

The high internal consistency of the total fidelity scale indicates that it is meaningful to use these as a measure on an evidence-based practice of physical health care. The correlation between the two subscales was strong, except at baseline where there was lower variance. But as shown in Fig. 1, the Practices fidelity is measured consistently lower than the Policies fidelity, as shown in many studies on Policies and Practices. This may indicate that Policies may influence Practices, but that making Policies are not enough to change the behavior of clinicians. In our study some of the difference between the measured fidelity of Policies and Practices may be due to differences in the calibration of items.

The significant increase in total scale fidelity suggests that the fidelity scale is sensitive to change and that it discriminates between sites with different levels of fidelity. The documented change occurred almost exclusively during the first six months. As the items with low ratings also after 18 months did not contribute much to the significant change, the change was determined mainly by changes in a little more than half of the items. As discussed above, it may be that the criteria are too strict for some of the items. This is also indicated by comments reported from the fidelity raters that some clinicians had felt that the great efforts they had put into some of the activities were not reflected in the fidelity ratings, or that the emphasis on written procedures in some criteria did not capture well-established non-written procedures at some sites.

The fidelity assessors found that the feasibility of the fidelity scale was good. However, an important finding is that it was significantly more difficult to find the information than to rate the items once they had found the information, and this was reported both for ratings based on interviews and in reading patient records. It was encouraging that the leaders reported that it was useful to get the feedback from the fidelity ratings, and that they reported that the fidelity ratings were used to improve antipsychotic medication management.

### Limitations

Several limitations warrant mention. The fidelity scale had minimal pilot testing. Some information was difficult to find in the patient records, perhaps especially regarding physical health care given by other agencies. Some ratings were not reliable, and the numbers of sites were low. Another limitation is that assessment and treatment of Hepatitis C was not included in the toolkit and the fidelity scale.

In our efforts to operationalize the content of each item of the fidelity scale we aimed to identify specific measurable criteria which could be reliably assessed as met or unmet. It is a challenge to establish quantitative criteria for fidelity items when the evidence is imprecise, and experts disagree. In many areas of medicine, researchers disagree on the benchmarks for performance. For the first version of this scale, we have used quantitative guidelines that are supported by some previous guidelines.

The fidelity of physical health care reported from the current study may not be representative for mental health services in other countries. There may be wide variations in this both across countries and within countries. But the fidelity scale should be able to measure to what extent evidence-based physical health care is given, and to guide efforts to improve or implement an evidence-based model of physical health care for people with psychosis.

## Conclusion and Implications

The Physical Health Care Fidelity Scale shows good to excellent interrater reliability, adequate sensitivity for change and good feasibility. It is the first fidelity scale for evidence-based physical health care for patients with serious mental illness. The fidelity scale can be used to measure and guide implementation of evidence-based physical health care reliably and with an acceptable use of time for clinicians and fidelity assessors.

## Electronic supplementary material

Below is the link to the electronic supplementary material.

Supplementary file1 (PDF 176 kb)

Supplementary file2 (PDF 60 kb)

Supplementary file3 (PDF 243 kb)
