# Supplementary material for: The Physical Health Care Fidelity Scale: Psychometric Properties
Source: Adm Policy Ment Health. 2020 Feb 8;47(6):901–10. doi: 10.1007/s10488-020-01019-0 (PMC7547955; doi:10.1007/s10488-020-01019-0)
Supplement: Supplementary file 1 — Supplementary file1 (PDF 176 kb) [file 10488_2020_1019_MOESM1_ESM.pdf]

Table 4* (online). Percentage exact agreement and interrater reliability for items and criteria: Independent pairs of rating of 13 sites at 4 times for items 1-6 and for 95 patient records for items 7-17**

#	Items (1-17) and criteria (a-e)	Agreement	ICC/Kappa**
	**Policies Subscale items and criteria**		
**1**	**Policy promoting physical fitness**	**65 %**	**.90**
a	Coordinator for fitness activities	87 %	.68
b	Fitness coordinator negotiates gym	84 %	.88
c	Physical activities are made known	90 %	.75
d	Help to track physical activities	94 %	.74
**2**	**Practical help to physical activities**	**81 %**	**.88**
a	Physical activity groups at least twice a week	96 %	.88
b	Physical fitness room or help to use a gym	92 %	.57
c	Exercise options for various patient preferences	90 %	.57
d	Exercise groups offered at suitable times	98 %	.88
**3**	**Policy supporting healthy diet**	**69 %**	**.88**
a	Policy and information on healthy diet	85 %	.68
b	No sugary beverages or junk food on premises	94 %	.74
c	Meals on premises are mainly nutritious	100 %	1.00
d	Unit has collaboration with a dietician	92 %	.73
e	Unit provides access to cooking classes	94 %	.79
**4**	**Policy supporting smoking cessation**	**62 %**	**.89**
a	Policy and information on no-smoking	92 %	.83
b	Smoking cessation regular topic in consultations	75 %	.47
c	Unit offers support for smoking cessation	87 %	.65
d	Nicotine patches or gums are accessible	93 %	.77
**5**	**Policy supporting dental health**	**79 %**	**.92**
a	Policy and information on dental health	88 %	.75
b	Procedures for monitoring dental health	94 %	.88
c	Toothbrush, toothpaste and dental floss available	96 %	.89
d	Assists appointment with dental hygienist or dentist	92 %	.46
**6**	**Collaboration and communication with GP**	**77 %**	**.85**
a	Policy of regular communication with GP and others involved	87 %	.51
b	Policy on regular information exchanged with GP	83 %	.65
c	Routine electronic transfer of information with GP and others	96 %	.85
d	Collaboration with a specialists or unit in internal medicine	98 %	.94
	**Practices Subscale items and criteria**		
**7**	**Support for regular physical activities**	**96 %**	**0.80**
a	Plan for physical activities documented in patient records	93 %	.76
b	Exercise plan approved by a physician	92 %	.67
c	Patient has been offered to join a group for physical activities	88 %	.75
d	Methods to track physical activities discussed with patient	96 %	.73
e	Review of exercises done by patient is documented	70 %	.39
**8**	**Monitoring of physical health conditions**	**93 %**	**.85**
a	Physical examinations documented in defined place in records	93 %	.85
b	Results of all laboratory or other tests easily accessible in	92 %	.75
c	No more than 6 months since last documented physical	88 %	.76
d	Follow up of physical health is planned, and it is followed up	77 %	.49
**9**	**Documented collaboration with GP**	**84 %**	**.66**

a	Communication with GP is documented in patient record	86 %	.72
b	Information from GP documented in a specified place in	85 %	.42
c	List of current medications in a defined place and easily	93 %	.63
d	Documented medication to GP after discharge or change	93 %	.85
e	Documented who is responsible for medical follow up	76 %	.51
**10**	**Documented support for healthy diets**	**96 %**	**.58**
a	Documented going through a full diet assessment with patient	95 %	.26
b	Documented staff suggesting healthy food choices for patient	90 %	.55
c	Documented help to join a cooking classes for healthy food	96 %	.58
**11**	**Monitoring BMI and waist circumference**	**99 %**	**.90**
a	Patient educated about BMI and overweight/underweight	97 %	.38
b	Weight recorded or attempted recorded each month or no	85 %	.64
c	BMI calculated and discussed with patient at least once	94 %	.72
d	Waist circumference has been measured at least once	96 %	.81
e	Weight change/stability discussed with patient	85 %	.62
f	Patient encouraged to track weight	97 %	.78
g	Patient receives recognition for reaching target weight	97 %	-.01
**12**	**Assessment and treatment of obesity or malnutrition**	**89 %**	**.64**
a	Assessment done whether patient is obese or at risk for obesity	76 %	.52
b	Assessment for sleep apnea has been done and documented	99 %	.66
c	Referred to specialist or GP within a month if high BMI	87 %	.68
d	If obesity, treatment for obesity is followed by unit, GP or	78 %	.44
e	Patient has met with dietician for overweight, or no obesity	87 %	.61
**13**	**Assessment and treatment of hypertension**	**93 %**	**.85**
a	Blood pressure documented measured at least once	98 %	.96
b	Treatment of hypertension followed up, or no hypertension	91 %	.81
c	Documented who is following blood pressure, or no	94 %	.87
**14**	**Assessment and regulation of blood sugar**	**89 %**	**.79**
a	Blood sugar measured at least once	95 %	.89
b	If medication or risk, blood sugar is measured monthly	89 %	.79
c	If elevated blood sugar, treatment followed by unit, GP or	91 %	.81
d	Documented who is following up blood sugar, or not elevated	87 %	.75
**15**	**Assessment and regulation of blood lipids**	**94 %**	**.87**
a	Blood lipids have been measured at least once	98 %	.96
b	If elevated blood lipids, treatment followed by unit, GP or	95 %	.89
c	Documented who is following up blood lipids, or not elevated	95 %	.89
**16**	**Interventions for smoking cessation**	**93 %**	**.66**
a	Patient has been asked about tobacco use	91 %	.81
b	If smoker, health risk has been discussed, or not smoking	89 %	.61
c	Educated about support for smoking cessation, or not smoking	95 %	.71
d	Smoking cessation program offered, including nicotine patches	94 %	.74
**17**	**Monitoring of dental health**	**98 %**	**.85**
a	Checkup/dental cleaning by dentist/dental hygienist at least	88 %	.67
b	Received information on oral hygiene in relation to medication	95 %	.73
c	Brushing twice daily and use of dental floss	98 %	.90
d	Application for free dentist has been sent or approved	91 %	.36
	**Summary for items (like in Table 2):**		
	**Average agreement and ICC for items 1-6 (Policies)**	**72 %**	**.89**
	**Average agreement and Kappa for items 6-17 (Practices)**	**93 %**	**.77**

	**Average agreement for all 17 items**		**86 %**	
	**Summaries for criteria:**			
	**Average for criteria of Policies items**		**91 %**	**.74**
	**Average for criteria of Practices items**		**91 %**	**.67**
	**Average for criteria of the total fidelity scale**		**91 %**	**.70**
	**Distribution of ratings of criteria**			
	**Exact agreement**	**Policies criteria**	**Prescriber Practices**	**Total fidelity scale**
	60- 69 %	0	0	0
	70-79 %	1	5	6
	80-89 %	7	12	19
	90-100 %	17	30	47
	**Cohen's Kappa**	**Policies criteria**	**Prescriber Practices**	**Total fidelity scale**
	Poor (< .21)	9	1	1
	Fair (.21 - .40)	9	4	4
	Moderate (.41 - .60)	5	8	13
	Substantial (.61 - .74)	7	14	21
	Excellent (.75 – 1.00)	13	20	33

*) Table 4 also contains the results for items reported in Table 2 in the paper, so that these here in Table 4 may be seen together with the results for the criteria.
**) ICC for items rated 1-5 and Cohen's Kappa for patient records rated passed/failed and for criteria rated passed/failed.
