# Supplementary material for: The Physical Health Care Fidelity Scale: Psychometric Properties
Source: Adm Policy Ment Health. 2020 Feb 8;47(6):901–10. doi: 10.1007/s10488-020-01019-0 (PMC7547955; doi:10.1007/s10488-020-01019-0)
Supplement: Supplementary file 2 — Supplementary file2 (PDF 60 kb) [file 10488_2020_1019_MOESM2_ESM.pdf]

Table 5 (online). Criteria passed for Policies items (Item 1-6) for 13 sites at 18 months, and patient records and criteria passed for 126 patient records reviewed at 13 sites at 18 months for Practices items (Item 7-17) **. Mean fidelity is reported in parenthesis for each item.

Items	Policies Subscale items and criteria	Failed	Passed
**1**	**Policy promoting physical fitness (3.23)**		
a	Coordinator for fitness activities	3	10
b	Fitness coordinator negotiates gym	9	4
c	Physical activities are made known	1	12
d	Help to track physical activities	10	3
**2**	**Practical help to physical activities (4.54)**		
a	Physical activity groups at least twice a week	2	11
b	Physical fitness room or help to use a gym	2	11
c	Exercise options for various patient preferences	1	12
d	Exercise groups offered at suitable times	1	12
**3**	**Policy supporting healthy diet (3.23)**		
a	Policy and information on healthy diet	2	11
b	No sugary beverages or junk food on premises	1	12
c	Meals on premises are mainly nutritious	0	13
d	Unit has collaboration with a dietician	10	3
e	Unit provides access to cooking classes	10	3
**4**	**Policy supporting smoking cessation (3.62)**		
a	Policy and information on no-smoking	4	9
b	Smoking cessation regular topic in consultations	4	9
c	Unit offers support for smoking cessation	7	6
d	Nicotine patches or gums are accessible	3	10
**5**	**Policy supporting dental health (4.08)**		
a	Policy and information on dental health	5	8
b	Procedures for monitoring dental health	4	9
c	Toothbrush, toothpaste and dental floss available	3	10
d	Assists appointment with dental hygienist or dentist	0	13
**6**	**Collaboration and communication with GP* (3.77)**		
a	Policy of regular communication with GP* and others involved	1	12
b	Policy on regular information exchanged with GP*	6	7
c	Routine electronic transfer of information with GP* and others	0	13
d	Collaboration with a specialists or unit in internal medicine	9	4
	**Practices Subscale items and criteria ****	**Failed**	**Passed**
**7**	**Support for regular physical activities (1.85)**	100	26
a	Plan for physical activities documented in patient records	93	33
b	Exercise plan approved by a physician	97	29
c	Patient has been offered to join a group for physical activities	85	41
d	Methods to track physical activities discussed with patient	115	11
e	Review of exercises done by patient is documented	62	64
**8**	**Monitoring of physical health conditions (3.31)**	61	65
a	Physical examinations documented in defined place in records	54	72
b	Results of laboratory or other tests easily accessible in records	18	108
c	No more than 6 months since last documented physical exam.	60	66
d	Follow up of physical health is planned, and it is followed up	82	44

**9**	**Documented collaboration with GP* (3.23)**	60	66
a	Communication with GP* is documented in patient record	40	86
b	Information from GP* documented in a specified place in	87	39
c	List of current medications in a defined place and easily	5	121
d	Documented medication to GP* after discharge or change	53	73
e	Documented who is responsible for medical follow up	50	76
**10**	**Documented support for healthy diets (1.46)**	108	18
a	Documented going through a full diet assessment with patient	116	10
b	Documented staff suggesting healthy food choices for patient	95	31
c	Documented help to join a cooking classes for healthy food	113	13
**11**	**Monitoring BMI and waist circumference (1.54)**	107	19
a	Patient educated about BMI and overweight/underweight	116	10
b	Weight recorded or attempted recorded each month or no	85	41
c	BMI calculated and discussed with patient at least once	103	23
d	Waist circumference has been measured at least once	106	20
e	Weight change/stability discussed with patient	91	35
f	Patient encouraged to track weight	100	26
g	Patient receives recognition for reaching target weight	113	13
**12**	**Assessment and treatment of obesity or malnutrition (1.77)**	97	29
a	Assessment done whether patient is obese or at risk for obesity	71	55
b	Assessment for sleep apnea has been done and documented	123	3
c	Referred to specialist or GP* within a month if high BMI	89	37
d	If obesity, treatment for obesity is followed by unit/GP*/others	85	41
e	Patient has met with dietician for overweight, or no obesity	96	30
**13**	**Assessment and treatment of hypertension 3.15)**	62	64
a	Blood pressure documented measured at least once	57	69
b	Treatment of hypertension followed up, or no hypertension	62	64
c	Documented who is following blood pressure, or no	62	64
**14**	**Assessment and regulation of blood sugar (3.92)**	46	80
a	Blood sugar measured at least once	38	88
b	If medication or risk, blood sugar is measured monthly	43	83
c	If elevated blood sugar, treatment followed by unit, GP* or	42	84
d	Documented who is following up blood sugar, or not elevated	49	77
**15**	**Assessment and regulation of blood lipids (3.00)**	65	61
a	Blood lipids have been measured at least once	61	65
b	If elevated blood lipids, treatment followed by unit, GP* or	62	64
c	Documented who is following up blood lipids, or not elevated	65	61
**16**	**Interventions for smoking cessation (1.62)**	102	24
a	Patient has been asked about tobacco use	71	55
b	If smoker, health risk has been discussed, or not smoking	98	28
c	Educated about support for smoking cessation, or not smoking	106	20
d	Smoking cessation program offered, including nicotine patches	102	24
**17**	**Monitoring of dental health (1.46)**	109	17
a	Checkup/dental cleaning by dentist/dental hygienist at least	98	28
b	Received information on oral hygiene in relation to medication	104	22
c	Brushing twice daily and use of dental floss	105	21
d	Application for free dentist has been sent or approved	109	17

*) GP = general practitioner. **) For each patient record the item was rated passed if all but one of the criteria were rated passed, except for item 11 (5 of 7 needed) and items 13 and 15 (all 3 criteria needed).
