# Supplementary material for: The Physical Health Care Fidelity Scale: Psychometric Properties
Source: Adm Policy Ment Health. 2020 Feb 8;47(6):901–10. doi: 10.1007/s10488-020-01019-0 (PMC7547955; doi:10.1007/s10488-020-01019-0)
Supplement: Supplementary file 3 — Supplementary file3 (PDF 243 kb) [file 10488_2020_1019_MOESM3_ESM.pdf]

The Physical Health Care Fidelity Scale

Clinical unit (site): ____ Completed at ____________ (date) by _________________________ (assessor)

Brief instructions

<u>Procedure for fidelity assessment</u>

- Fidelity assessment is done during one day by two fidelity assessors who are not working in the unit.
- The fidelity assessors have been trained in using the fidelity scale with the instructions and summary sheets for the scale.
- The fidelity assessment is prepared and done as described in the instructions for preparation and implementation of the assessment.

<u>Preparations for the fidelity assessment</u>

- The unit must be clearly defined regarding which teams/services that are included in the unit and the fidelity assessment.
- The unit manager prepares the program for the day in collaboration with the assessor from the health trust.
- The unit manager identifies all relevant documents/procedures and send these to the assessor before the assessment.
- The unit manager makes arrangement with managers, clinicians and others for interviews during the assessment day.
- The unit manager ensures that print out of 10 randomly selected patient records are available as specified in the instructions for preparation and implementation of the assessment.

<u>Tools for the fidelity assessment</u>

- The fidelity scale with criteria (this document).
- Instructions and interview guide for fidelity assessment of physical health care at the system level (elements/items 1-6).
- Summary sheet for review and rating of patient records for fidelity assessment of physical health care at the individual level (elements/items 7-17).

The Physical Health Care Fidelity Scale – Page 2

Elements of practice	Rating and criteria met	Sources of information	Criteria for fidelity measure and rating

Items of the Policies Subscale (use interview guide and form)				
**1**	**The unit has a policy (established practice) and staffing promoting physical fitness**		Written material, observations, interviews with staff and patients	a. At least one staff is coordinator for fitness activities with a specified allocated number of hours each week with job duties to facilitate physical fitness (lead exercise groups, identify fitness centers and other options in the community).<br>b. Fitness coordinator negotiates arrangements with at least one community gym/fitness center for free memberships or discounted rates.<br>c. Unit make physical activities known by brochures, posters, newsletters or announcements. Every new patient receives a brochure on physical activities and a personal welcome to participate.<br>d. Unit offers pedometers and/or instructions for using apps to track physical acticites.<br>**Scoring: Each criterion scored yes/no. Fidelity item score = sum of yeses + 1**
**2**	**The unit offer practical help to physical activites**		Written material, observations, interviews with staff and patients	a. Unit offers physical activity groups at least twice a week.<br>b. Unit has a physical fitness room with training equipment for patients, and staff can show the patients how to use the equipment, or the unit helps patients to contact and use a gym/fitness center in the community.<br>c. Two or more other exercise options are available for a variety of patient preferences.<br>d. Exercise groups are offered at times that most patients can participate.<br>**Scoring: Each criterion scored yes/no. Fidelity item score = sum of yeses + 1**
**3**	**The unit has a policy (established practice) supporting healthy diet**		Written material, observations, interviews with staff and patients	a. Unit has a policy (established practice) of healthy diet and posters and/or brochures about choosing healthy food choices, and it may give information on websites.<br>b. No sugary beverages or junk food in vending machine on premises.<br>c. Meals served on premises are mainly nutritious, or no meals are served.<br>d. The unit has established collaboration with a dietician or has dietician in staff.<br>e. Unit provides or helps access to cooking classes focusing healthy food.<br>**Scoring: Each criterion scored yes/no. Fidelity item score = sum of yeses (0 yeses is rated as 1)**

The Physical Health Care Fidelity Scale – Page 3

	Elements of practice	Rating and criteria met	Sources of information	Criteria for fidelity measure and rating
**4**	**The unit has policies to support smoking cessation**		Written material, observations, interviews with staff and patients	a. The unit has a policy (established practice) of no-smoking and posters/brochures on help to stop smoking.<br>b. Smoking and smoking cessation are regular topics in assessment and documentation.<br>c. Unit offers or helps access to smoking cessation groups or individual support by telephone or internet to stop smoking.<br>d. Nicotine patches or gums are accessible as help to stop smoking.<br>**Scoring: Each criterion scored yes/no. Fidelity item score = sum of yeses + 1**
**5**	**The unit has policies to support dental health**		Written material, observations, interviews with staff and patients	a. The unit has a policy (established practice) of dental health and posters/brochures on dental health.<br>b. The unit has procedures for how staff should monitor patient dental health.<br>c. The unit offers toothbrush, toothpaste and possibly dental floss to all patients who do not have it.<br>d. The unit actively assists to arrange appointment with a dental hygienist or dentist, if patients do not have regular appointments.<br>**Scoring: Each criterion scored yes/no. Fidelity item score = sum of yeses + 1**
**6**	**Active collaboration and communication between unit and general practitioners**		Written material, observations, interviews with staff and patients	a. The unit has a policy (established practice) that ensures regular communication with GP and other medical personnel that the patient has contact with outside the unit.<br>b. The unit policy (established practice) specifies how often information should be exchanged with GP during an ongoing treatment episode of a patient.<br>c. The unit has established procedures for routine electronic transfer of necessary information on physical health and measures to and from GP and others involved in following up the physical health of the patient.<br>d. The unit has established collaboration with a specialists or unit in internal medicine<br>**Scoring: Each criterion scored yes/no. Fidelity item score = sum of yeses + 1**

	Elements of practice	Rating and criteria met	Sources of information	Criteria for fidelity measure and rating
**Items of the Practices Subscale (use summary sheet for review of patient records)**				
**7**	**Documented support for regular physical activities**		Patient records and charts	***Patient medical records include documentation of:***<br>o A specified plan for physical activities is documented in patient records (e.g. goals, what type, what time of day, with whom).<br>o A physician has approved that the patient may follow the exercise plan.<br>o Patient has been offered to join a group for physical activities.<br>o Individualized method to track physical activities have been discussed with patient.<br>o Review of exercises done by patient is documented in at least one note.<br><br>**Procedure: Chart rated pass if at least 4 of the 5 criteria above are met.**<br><br>**Scoring: ≥ 8 charts pass = 5, 6-7 charts= 4, 4-5 charts = 3, 2-3 charts = 2, 0-1 charts = 1.**
**8**	**Monitoring of physical health conditions**		Patient records and charts	***Patient medical records include documentation of:***<br>o Physical examinations are documented in a defined place in patient records.<br>o Results of all laboratory or other tests are easily accessible in records.<br>o No more than 6 months since last documented physical examination.<br>o Follow up of physical health is planned, and it is followed up.<br><br>**Procedure: Chart rated pass if at least 3 of the 4 criteria above are met.**<br><br>**Scoring: ≥ 8 charts pass = 5, 6-7 charts= 4, 4-5 charts = 3, 2-3 charts = 2, 0-1 charts = 1.**

Elements of practice		Rating and criteria met	Sources of information	Criteria for fidelity measure and rating
**9**	**Documented collaboration with GP and other medical professionals regarding physical health**		Patient records and charts	***Patient medical records include documentation of:***<br>○ Communication between unit clinician and GP is documented in patient record.<br>○ Information from GP on physical health (examinations, tests, plans, treatment) is documented in a specified place in records and is easily available.<br>○ List of current medications is kept in a defined place and is easily available.<br>○ Documentation that list of medications is sent to GP within a week after discharge (discharge letter) or (for outpatients) after change of medication.<br>○ Documentation of who is responsible for follow up on each medical condition, or no medical condition to follow up.<br><br>**Procedure: Chart rated pass if at least 4 of the 5 criteria above are met.**<br><br>**Scoring: ≥ 8 charts pass = 5, 6-7 charts= 4, 4-5 charts = 3, 2-3 charts = 2, 0-1 charts = 1.**
**10**	**Documented support for healthy diets**		Patient records and charts	***Patient medical records include documentation of:***<br>○ Documented going through a full diet assessment (24 hours diet, including beverages and snacks) with patient.<br>○ Documentation of staff suggesting healthy food choices for shopping and for preparing meals.<br>○ Documentation that cooking classes for healthy food are offered, or that patient is helped to join such classes elsewhere.<br><br>**Procedure: Chart rated pass if at least 2 of the 3 criteria above are met.**<br><br>**Scoring: ≥ 8 charts pass = 5, 6-7 charts= 4, 4-5 charts = 3, 2-3 charts = 2, 0-1 charts = 1.**

	Elements of practice	Rating and criteria met	Sources of information	Criteria for fidelity measure and rating
**11**	**Monitoring BMI and waist circumference**		Patient records and charts	***Patient records include documentation of following activities by unit or GP:***<br>○ Patient is educated about BMI, BMI cutoff for overweight/underweight, and health threats of obesity/underweight.<br>○ Weight recorded or attempted recorded each month, or no obesity.<br>○ BMI calculated and discussed with patient at least once.<br>○ Waist circumference has been measured at least once.<br>○ Weight change/stability discussed with patient at least once.<br>○ Patient encouraged to track weight.<br>○ Patient receives recognition for reaching target weight milestones.<br><br>**<u>Procedure</u>: Chart rated pass if at least 5 of the 7 criteria above are met.**<br><br>**<u>Scoring</u>: ≥ 8 charts pass = 5, 6-7 charts= 4, 4-5 charts = 3, 2-3 charts = 2, 0-1 charts = 1.**
**12**	**Assessment and treatment of obesity or malnutrition**		Patient records and charts (see instruction for extraction of records)	***Patient records include documentation of:***<br>○ Assessment has been done whether patient is obese or at risk for obesity.<br>○ Assessment for sleep apnea has been done and documented.<br>○ Patient has been referred to specialist or GP within a month if BMI ≥ 40 or BMI ≥ 35 + comorbidity (diabetes, hypertension, sleep apnea), or not problem of obesity.<br>○ If obesity, treatment for obesity is followed by unit, GP or others.<br>○ Patient has met with dietician (individually or in group) as part of treatment of overweight conditions, or no obesity<br><br>**<u>Procedure</u>: Chart rated pass if at least 4 of the 5 criteria above are met.**<br><br>**<u>Scoring</u>: ≥ 8 charts pass = 5, 6-7 charts= 4, 4-5 charts = 3, 2-3 charts = 2, 0-1 charts = 1.**

	Elements of practice	Rating and criteria met	Sources of information	Criteria for fidelity measure and rating
**13**	**Assessment and treatment of hypertension**		Patient records and charts	***Patient medical records include documentation of:***<br>○ Blood pressure documented measured at least once.<br>○ If elevated blood pressure, treatment of hypertension is followed up by unit, GP or others, or no hypertension.<br>○ If patient is followed by several agencies, it is documented who is following up elevated blood pressure, or no hypertension.<br><br>**Procedure: Chart rated pass if all 3 criteria above are met.**<br>**Scoring: ≥ 8 charts pass = 5, 6-7 charts= 4, 4-5 charts = 3, 2-3 charts = 2, 0-1 charts = 1.**
**14**	**Assessment and regulation of blood sugar**		Patient records and charts	***Patient medical records include documentation of:***<br>○ Blood sugar measured at least once.<br>○ If medication or risk, blood sugar is measured monthly, or not elevated blood sugar.<br>○ If elevated blood sugar, treatment of this is followed by unit, GP or others.<br>○ If patient is followed by several agencies, it is documented who is following up elevated blood sugar, or no elevated blood sugar.<br><br>**Procedure: Chart rated pass if at least 3 of the 4 criteria above are met.**<br>**Scoring: ≥ 8 charts pass = 5, 6-7 charts= 4, 4-5 charts = 3, 2-3 charts = 2, 0-1 charts = 1.**
**15**	**Assessment and regulation of blood lipids**		Patient records and charts	***Patient medical records include documentation of:***<br>○ Blood lipids have been measured at least once.<br>○ If elevated blood lipids, treatment of this is followed by unit, GP or others.<br>○ If patient is followed by several agencies, it is documented who is following up elevated blood lipids, or no elevated blood lipids.<br><br>**Procedure: Chart rated pass if all 3 criteria above are met.**<br>**Scoring: ≥ 8 charts pass = 5, 6-7 charts= 4, 4-5 charts = 3, 2-3 charts = 2, 0-1 charts = 1**

	Elements of practice	Rating and criteria met	Sources of information	Criteria for fidelity measure and rating
**16**	**Interventions for smoking cessation**		Patient records and charts (ask for additional records/charts until 5 smokers assessed)	***Patient medical records include documentation of:***<br>○ Patient has been asked about tobacco use.<br>○ If smoker, health risk has been discussed, - or patient is not smoking.<br>○ Patient has been educated about app for smoking cessation and about website supporting smoking cessation, - or patient is not smoking.<br>○ Smoking cessation program is offered, including nicotine patches/gum, - or patient is not smoking.<br><br>**<u>Procedure</u>: Chart rated pass if at least 3 of the 4 criteria above are met.**<br>**<u>Scoring</u>: ≥ 8 charts pass = 5, 6-7 charts= 4, 4-5 charts = 3, 2-3 charts = 2, 0-1 charts = 1.**
**17**	**Monitoring of dental health**		Patient records and charts	***Patient medical records include documentation of:***<br>○ Patient has checkup and dental cleaning by dentist or dental hygienist at least once.<br>○ Patient has received written and oral information on the importance of oral hygiene and brushing in relation to medication.<br>○ Brushing twice daily and use of dental floss/tooth sticks has been asked/checked.<br>○ Application for free dentist has been sent (or approved).<br><br>**<u>Procedure</u>: Chart rated pass if at least 3 of the 4 criteria above are met.**<br>**<u>Scoring</u>: ≥ 8 charts pass = 5, 6-7 charts= 4, 4-5 charts = 3, 2-3 charts = 2, 0-1 charts = 1.**
